# Branchless distance-driven and hybrid projectors in iterative cone beam CT

**DOI:** 10.1177/08953996261433875

**Published:** 2026-04-13

**Authors:** Ville-Veikko Wettenhovi, Ari Hietanen, Nargiza Djurabekova, Kati Niinimäki, Marko Vauhkonen, Ville Kolehmainen

**Affiliations:** 1 Department of Technical Physics, University of Eastern Finland, Finland; 2 Planmeca Group, Helsinki, Finland

**Keywords:** cone beam computed tomography, image reconstruction, model-based iterative, projector, distance-driven

## Abstract

**Purpose:**

Iterative model-based image reconstruction algorithms in cone beam computed tomography (CBCT) require repetitive forward and backward projection operations. We compare the quality of the branchless distance-driven (BDD) projector in iterative CBCT reconstruction with ray- and voxel-based methods in both regular and low-dose examinations, and introduce a hybrid approach that aims at faster computation by using the BDD as the backprojector only. We also demonstrate the potential of the BDD in FDK reconstructions.

**Approach:**

Two measured and one simulated datasets are used. Contrast-to-noise ratio (CNR) and modulation transfer function values are computed for one measured dataset. The structural similarity index (SSIM) and peak signal-to-noise ratio (PSNR) are computed with the simulated data.

**Results:**

Based on our results, BDD has reduced noise and better CNR compared to the other methods, with the quality dependent on the scanner geometry. The CNR is improved by 21% with the BDD and 4% with the hybrid method. BDD improves SSIM values by approximately 3.3% in the lowest dose case and 1% in the highest dose case, while for PSNR the values are 5% - 10% better. For the hybrid method, the SSIM improvements range from 0.8% - 2.2%, and the PSNR from 3.7% - 6.6 %. The hybrid method with the BDD as a backprojector can be computationally twice faster with similar image quality.

**Conclusions:**

The hybrid projector is a good choice as a compromise between image quality and computation time. Furthermore, BDD and the hybrid projector are better choices in low-dose CBCT reconstructions.

## Introduction

X-ray computed tomography (CT) is a widely used imaging modality for the non-invasive and non-destructive investigation of structures. While CT is most commonly associated with medical imaging applications, it is also used in industrial imaging,^1,2^ examination of archaeological objects,^3,4^ geological studies,^5^ as well as in security screening.^6,7^ CT allows the examination of an object’s structure based on the material’s attenuating properties. CT can also achieve high spatial resolution, especially when utilizing µCT, and fast scan times, when compared to, for example, magnetic resonance imaging.

Modern medical CT-based imaging methods can be divided into two different subcategories: helical CT and cone beam CT (CBCT). Helical CT is widely used in hospital settings as the standard CT for whole-body imaging. CBCT, on the other hand, is used in more specialized scanning applications, often when a higher spatial resolution is needed without increasing the radiation dose. Such applications include dental imaging, radiotherapy planning, imaging of the head, and imaging of extremities. While CBCT can be used in similar applications to those of helical CT, the two methods currently have somewhat distinct areas of use.

The advantages of CBCT over helical CT are a lower dose and the ability to perform focused scanning. Since the patient, and often the detector panel, can be moved closer to or farther from the source, CBCT allows directing the X-rays to scan a specific region of interest (ROI). Although the same can be achieved with fixed helical scanners, this requires collimation and thus reduces the usable region of the detector panel. While CBCT generally already uses small doses, focused scanning can help reduce the dose even further as tissues outside the ROI are exposed in only part of the projections, making the exposure minimal outside the ROI.

The image reconstruction processes of both helical and CBCT are similar. As such, the same methods can be utilized for both, with some differences due to different scanner designs or scan types, such as offset imaging.^8^ The core components of any CT image reconstruction are the projectors, namely the forward and backward projection operations. Forward projection transforms the image into projections, i.e. from image to measurement space. Backprojection operates backward by transforming projections into images, from measurement to image space. These projection operations are based on the geometrical model of the system and can be roughly divided into four distinct categories: ray-driven, voxel-driven, volume and area-driven, and distance-driven.^9–12^ When selecting a projector, the key aspects are the image quality and computational cost involved. Although a certain projector might provide a more optimal image quality, this might come at a high computational cost, making the projector impractical in applications where fast reconstruction is required.

Ray-driven projectors model the geometry by projecting a ray from the source to a point on the detector and going through each source-detector combination, i.e. each measurement. Since the ray-driven projector operates in the measurement space, it is well-suited for forward projection, both in terms of computational efficiency and image quality. However, in backprojection, ray-driven methods can suffer from Moiré patterns^9^ due to an insufficient sampling rate. Furthermore, since the ray-driven projector operates in the measurement space, parallelizing the backprojection becomes suboptimal. Different threads may access the same voxel simultaneously, leading to either reduced accuracy or significantly diminished performance,^13^ making ray-driven projectors non-optimal choice for backprojection both in terms of quality and efficiency. As such, ray-driven methods are the most widely used forward projectors.

Voxel-based methods work similarly to ray-based ones, but instead of projecting the ray directly from the source to each detector element, the ray trajectory is determined based on the voxel position. Specifically, the ray from the source is projected through the center of a voxel and onto the detector panel. Since voxel-based methods operate in the image space, they are well-suited for backprojection, again both in terms of computational efficiency and image quality. However, like ray-based approaches in backprojection, voxel-based methods can suffer from Moiré patterns and be inefficient for forward projection.^9,14^ While there are ways to overcome these issues when computing the forward projection with voxel-based methods,^15–17^ they increase computational costs and thus sacrifice computational speed, making ray-based approaches favorable for forward projection computation.

The volume and area-driven models compute either the volume of intersection^18,19^ or the area of intersection^20^ of the cone beam and a voxel. These models are computationally very demanding. Additionally, like ray-driven methods, they operate in the measurement space, making them inefficient for backprojection.

The distance-driven methods, such as the distance-driven projector^9,21^ or the separable footprints projector,^12^ approximate the area of overlap between the voxel projections and the detector bins instead of accurately computing it. In the distance-driven projector, this is achieved by computing the rectangular area in the image- or measurement-space while the separable footprints projector uses a computationally slower but more accurate assumption of a trapezoid. Distance-driven methods are computationally more demanding than ray- or voxel-driven ones, but since they can be efficiently implemented on graphics processing units (GPUs)^22–25^ the computational time difference is not as significant. In this work, we focus on a GPU-optimized distance-driven method based on the branchless distance-driven (BDD) algorithm,^26^ where the branching of the original distance-driven algorithm^9^ is reduced to improve performance on GPUs. The BDD, however, can be as much as twice slower than the combination of a ray-based forward projector and a voxel-based backprojector even when optimized for GPU computing.

Classical closed-form reconstruction methods, such as the Feldkamp-Davis-Kress (FDK) algorithm,^27^ require only a single backprojection step to compute the image. In contrast, an emerging class of model-based iterative reconstruction algorithms formulates the reconstruction process as the optimization of a data fidelity functional, such as least squares (LS), often augmented with some regularization to promote prior information about the unknown image. These algorithms require several calls of the forward and backward projection operations to compute the reconstruction. Despite their good performance and flexibility in relation to scanning geometry and physics modeling, model-based iterative algorithms are not yet actively utilized in clinical CBCT because of their high computational cost. To study the performance of different projectors for iterative CBCT reconstruction, we use the primal-dual hybrid gradient (PDHG) algorithm^28,29^ for the minimization of the unregularized LS residual and the Condat-Vũ (CV)^30,31^ variant of the PDHG algorithm for regularized least squares with non-local means regularization.^32^

In our previous conference paper,^33^ we visually compared the BDD projector with a ray-driven forward projection and a voxel-based backprojection, as well as ray-driven forward and backward projection with data from one phantom. In this work, we expand on this topic, presenting three distinct studies. Furthermore, in this work, we solely focus on an interpolation-based forward projector and do not consider Siddon’s method that was also shown in our previous paper.^33^ This is because on a GPU the interpolation-based projector is faster than Siddon’s method but offers practically identical image quality.

First, we examine the usability of BDD in iterative model-based CBCT reconstruction, by minimizing the LS functional and regularized LS using both standard- and low-dose CBCT data. Second, we implement a hybrid projector that uses a ray-based forward projector and a BDD backprojector. Although the use of unmatched projectors in general is not novel, it has been studied in various imaging domains. For CT, the combination of a ray-driven forward projection with a voxel-based backprojector has been studied in.^34–37^ The use of mismatched projectors has also been investigated in emission tomography^38–40^ and microscopy.^41^ The convergence properties of image reconstruction algorithms in the presence of unmatched projectors and the modifications of the algorithms to guarantee convergence with unmatched projectors have been developed in.^42–46^ However, to the best of our knowledge, BDD has not been previously tested in this hybrid form.

The closest was a study from,^47^ where the original distance-driven projector^9^ was compared with Joseph’s method^48^ and a bilinear interpolation method. However, this study^47^ used 2D fan-beam geometry only on simulated data and utilized the unoptimized versions of the projectors, such as the original distance-driven instead of BDD. As the focus was on forward projection, there was no consideration of hybrid methods. In this work, all these concerns are addressed by using 3D cone beam data with the resolution and data size of real-world applications, presenting results from both simulated and measured CBCT data, utilizing much more computationally optimized methods specifically developed for GPU computing, and investigating hybrid approaches for the optimal ratio of computation time and image quality.

The paper is organized as follows. In Section II, we outline the algorithms for the projectors used in this study, namely the ray- and voxel-based approaches and the BDD. In Section III, we present the PDHG and CV algorithms, and the basic concepts behind non-local means regularization. In Section IV, we present the measurement setup and details on the selected parameters. In Section V, we present the results and in Section VI, we give a discussion and conclusions regarding the findings of this paper.

## Projectors

Throughout the paper, bold uppercase variables A denote matrices, bold lowercase variables a denote vectors, and regular lowercase, or uppercase, variables a denote scalars.

### Ray- and voxel-driven methods

Both the ray- and voxel-driven approaches presented here are based on linear interpolation. The combination of the ray- and voxel-based projections is thus referred to as the interpolation-based projector.

#### Forward projection

In ray-driven methods, we project a ray from the source to each detector pixel. The lines of intersection, i.e. the distance each ray traverses in a voxel, are computed, then multiplied with the voxel values in the image, and summed together to form the forward projection for each detector pixel. This process is called ray-tracing. For ray-driven methods, the core component is thus the computation of the line of intersection inside the image volume for each ray. For computing the accurate line of intersection for each voxel, a widely used method is the original Siddon method,^49^ which has subsequently received computationally more efficient versions.^50,51^

An alternative method is to interpolate the voxel values at given interpolation lengths, similar to Joseph’s method. Traditional CPU-based approaches would need a software implementation of 3D interpolation to compute these projectors, but with GPUs, this can be achieved automatically by using hardware interpolation. In OpenCL^52^ and SYCL,^53^ this is achieved by using images, while in CUDA, similar functionality exists with texture objects. With GPUs, the interpolation can thus be performed by simply providing the x , y , and z coordinates and choosing either linear or nearest-neighbor interpolation, i.e. hardware-based interpolation. Normalizing each coordinate x , y , and z to the [0,1] range can be beneficial for the interpolation, particularly when working with images or textures where 0 and 1 correspond to the boundaries along each axis. In ray-based projection methods that rely on interpolation, linear interpolation should be preferred as it can leverage hardware-accelerated texture sampling. Alternative interpolation methods (e.g. cubic or spline based) are possible, but these typically cannot utilize hardware interpolation and are often slower and less efficient.

Let $d\in R^{3}$ be the three coordinates for the center of the detector panel. Similarly, let $s\in R^{3}$ be the vector of coordinates of the source and $b_{\min}\in R^{3}$ the coordinates of the minimum point of the FOV, i.e. the corner where lower coordinate values would lead outside the FOV. Similarly, let $b_{\max}\in R^{3}$ be the maximum point where larger coordinates would lead outside the FOV. We use the parametric form of a line to represent the line from the source to the detector panel, i.e. $l=l_{a}+l_{ab}t$ , where $l\in R^{3}$ are the coordinates on the line at a specific point, $l_{a}\in R^{3}$ is a point along the line, $l_{ab}\in R^{3}$ is the direction vector from point a to point b and t∈R represents the position along the ray. In our case, $l_{a}=s$ and $l_{ab}=d-s$ . We need to determine the parameters $t_{\mathrm{start}}$ and $t_{\mathrm{end}}$ , which are the parameters for the coordinates when the ray enters the FOV and leaves the FOV, respectively. To do that, we first compute the parameters for the ray at the edges of the FOV and then use these to compute the starting and ending parameters:

(1) $$t_{\mathrm{front}}=\frac{\left(b_{\max}-s\right)}{l_{ab}}$$

(2) $$t_{\mathrm{back}}=\frac{\left(b_{\min}-s\right)}{l_{ab}}$$

(3) $$t_{\min}=\min(t_{\mathrm{front}},t_{\mathrm{back}})$$

(4) $$t_{\max}=\max(t_{\mathrm{front}},t_{\mathrm{back}})$$

(5) $$t_{\mathrm{start}}=\max(t_{\min,x},t_{\min,y},t_{\min,z})$$

(6) $$t_{\mathrm{end}}=\min(t_{\max},t_{\max,y},t_{\max,z}),$$

where $t_{\mathrm{front}}$ and $t_{\mathrm{back}}$ refer to the parameter values along the ray where it intersects with the FOV. Meanwhile, $t_{.,x/y/z}$ refers to the x/y/z -axis component of the min/max variables. All divisions are done elementwise. The step size $t_{\mathrm{step}}$ is obtained simply by dividing the desired interpolation length by the length of the ray. If $t_{\mathrm{start}}>t_{\mathrm{end}}$ , then the ray does not intersect with the FOV, and therefore no contribution is made to the projection. Otherwise, the ray traversal begins at $t_{\mathrm{start}}$ . At each step, the current position is computed using the parametric line equation, and linear interpolation is performed at that point. The interpolated value is multiplied by the interpolation length, which serves as a weight representing the segment of the ray that contributes to the projection. Next, we increment $t_{\mathrm{start}}$ by $t_{\mathrm{step}}$ and compute the new coordinates l . This process is repeated until $t>t_{\mathrm{end}}$ and iteratively accumulating the previous value at each step gives an approximation of the line-integral along the ray through the volume.

The benefit of the interpolation-based method is that one can freely choose the interpolation length. Longer interpolation lengths lead to faster computation at the expense of lower accuracy of the projector. Interpolation lengths of 0.5 to 1 times the voxel length are usually safe choices for sufficient accuracy, but larger ones may also lead to good reconstruction accuracy. The computational benefit, however, decreases rapidly after 2 times the voxel length. Computationally, the interpolation length of one voxel largely corresponds to an accurate modeling of the intersection length.

#### Backprojection

As previously mentioned, for backprojection, voxel-based projectors perform better than ray-based projectors. In voxel-based backprojection, the principle is to project a ray from the source through the center of each voxel onto the detector panel. The data value to be backprojected is obtained by finding the point of intersection between the ray and the detector panel. The value of the projection image at the point of intersection is calculated with nearest-neighbor or linear interpolation. Based on our tests, using linear interpolation can lead to slightly less noisy images than the nearest neighbor. The point of intersection can be obtained by computing the intersection point between the ray and the detector panel.

A point on the plane can be described as $m=m_{0}+m_{01}u+m_{02}v$ ( u,v∈R ), where 0, 1 and 2 are any points on the plane that form a triangle, and 01 and 02 indicate the vectors between the points. u and v are the parametric values of the plane. Now m=l and we get

(7) $$l_{a}+l_{ab}t=m_{0}+m_{01}u+m_{02}v,$$

(8) $$l_{a}-m_{0}=m_{01}u+m_{02}v-l_{ab}t$$

(9) $$\left[\begin{array}{cc} l_{a} & -m_{0} \end{array}\right]=\left[\begin{array}{ccc} -l_{ab} & m_{01} & m_{02} \end{array}\right]\left[\begin{array}{c} t\\ u\\ v \end{array}\right]$$

Taking the inverse in (9), we get

(10) $$\left[\begin{array}{c} t\\ u\\ v \end{array}\right]={\left[\begin{array}{ccc} -l_{ab} & m_{01} & m_{02} \end{array}\right]}^{-1}\left[\begin{array}{c} l_{a}-m_{0} \end{array}\right]$$

(11) $$\begin{array}{r} \left[\begin{array}{c} t\\ u\\ v \end{array}\right]=\frac{1}{-l_{ab}\cdot (m_{01}\times m_{02})}\left[\begin{array}{c} (m_{01}\times m_{02}{)}^{T}\\ (m_{02}\times l_{ab}{)}^{T}\\ (m_{01}\times l_{ab}{)}^{T} \end{array}\right]\left[l_{ab}-m_{0}\right] \end{array}$$

Solving for t gives

(12) $$t=\frac{\left(m_{01}\times m_{02})\cdot (l_{a}-m_{0}\right)}{-l_{ab}\cdot (m_{01}\times m_{02})},$$

where ⋅ is the dot product and × is the cross product. The coordinate is again normalized between [0,1], i.e. the borders of the detector panel. Thus, rays outside the panel are ignored.

If different kinds of projectors are used for the forward and backward projections, the projectors are usually not exactly adjoint. When using a ray-based forward projector with a voxel-based backprojector, such as the interpolation-based method, this can be alleviated by using a specific weight, or line of intersection, as described in^35^ for the voxel-based backprojector. The weight is

(13) $$w_{1}=\frac{\Delta x\Delta y\Delta zL^{3}}{\Delta r\Delta cL_{0}l^{2}},$$

where Δx/y/z are voxel sizes in each dimension, Δc/r are the width and height of each detector pixel, L is the distance from the source to the projected point on the detector, $L_{0}$ is the source-to-detector-distance and l is the distance from the source to the current voxel. The weight is thus voxel-specific. Multiplying the weight $w_{1}$ in (13) by the interpolated value, we obtain an element of the backprojection ( $A^{T}y$ ). Using this information, we can perform the adjointness test with

(14) $$(A\;f{)}^{T}y={\;f}^{T}(A^{T}y).$$

This makes the difference between the right- and left-handed sides less than 1%, according to our tests. In (14), A is the system matrix that models the system, f is a non-zero vector in the image space, and y a non-zero vector in the measurement space.

### Branchless distance-driven projector

**Figure 1.**
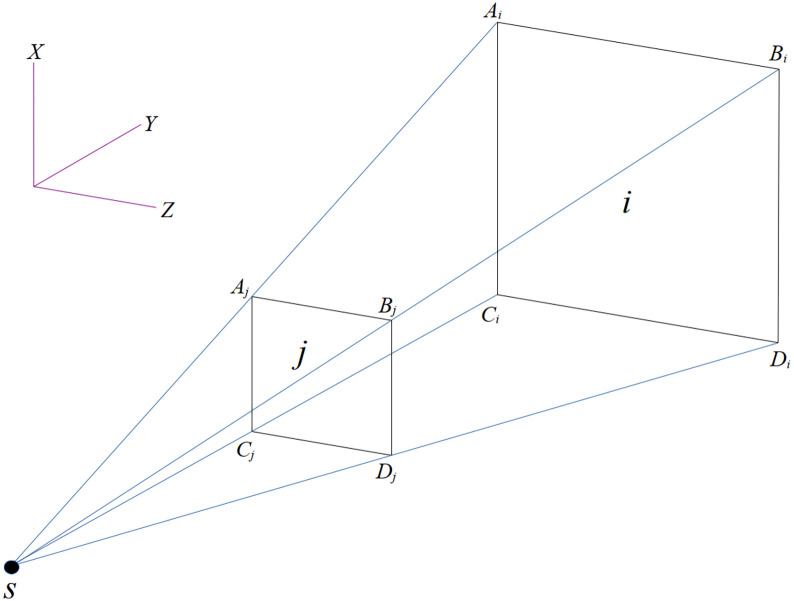
Illustration of the general principle of the BDD projector. S is the source, j a region in the image volume and i a region in the detector plane. A , B , C and D are the corners of the region.

The general idea of the branchless distance-driven (BDD) projector is outlined in Figure 1. Points on the detector plane in Figure 1 are preceded by i , while points in the image volume are preceded by j . In BDD, we project four rays from the source to either the corners of each detector pixel i (forward projection) or the central slice of each voxel j (backprojection). The goal is to compute the area enclosed by these rays in both cases, i.e. either the region i (backprojection) or region j (forward projection) in Figure 1. In both scenarios, the projections can be interpreted as shadows: in forward projection, the area of a single detector pixel represents a shadow of the image region area enclosed by the four rays; in backprojection, the voxel casts a shadow onto the detector panel. For forward projection, the size of the image region depends on the detector pixel size and its distance from the source, while in backprojection, the shadow size depends on the size of the voxel and its distance to the panel.

A straightforward way to compute the area of either the image region in forward projection or the shadow in backprojection is to precompute the integral image, also known as a summed-area table, of either the image volume or the projection image. By precomputing the cumulative sums of the input image, along both row and column dimensions, ahead of time, one can efficiently evaluate the sum in any rectangular region using only simple arithmetic operations.^54^ Details of this are presented below for forward and backward projections.

Since BDD relies on integral images to compute areas over rectangular regions, it assumes axis-aligned geometry. This can potentially lead to inaccuracies with CBCT as small rotation along the axis of the detector panel, such as roll or tilt, can occur because of the scanner structure.

In such cases, the BDD is not completely accurate. However, in all our test cases, this limitation was negligible. The observed geometric inaccuracy was not significant as the rotation was typically less than a 1-degree rotation on the XZ-plane and XY-plane.

Another potential disadvantage of using integral images is the possible loss of precision, especially when using single-precision (32-bit) values. As the integral image is the cumulative sum in both row and column directions, the values and the dynamic range can get large, and thus lose accuracy if the distance between the interpolated points is short. One way to reduce the dynamic range is to subtract the DC-component, i.e. the mean value, before computing the integral image and then add it back after obtaining the value for the area. While this was implemented, it did not appear necessary, as in all our test cases, there were no issues with the dynamic range.

Computing the integral image efficiently can be computationally challenging on GPUs due to the cumulative sums and the difficulty in parallelizing them. Although different algorithms exist for the computation of the integral image,^55,56^ we used the integral image function from the ArrayFire library.^57^

#### Forward projection

The forward projection of the BDD requires two different branches, depending on whether the ray first intersects the XZ -plane or the YZ -plane in the image volume (see Figure 1). Although the XY -plane may be relevant in some contexts, it is not considered here. As illustrated in Figure 1, we need to calculate the area enclosed by the points $A_{j}$ , $B_{j}$ , $C_{j}$ , and $D_{j}$ . For this, we need the integral images for each slice for the XZ - and YZ -planes. BDD forward projection thus requires the memory of two additional image volumes when compared to ray-based approaches. The increase in memory usage can be problematic if large image volumes are used, as is common in CBCT. The area for the region enclosed by the four rays can be computed from the integral images with

(15) $$Area_{f}=A_{j}+D_{j}-B_{j}-C_{j},$$

where each point j is linearly interpolated from the integral image. This is again efficiently computed on the GPU by using hardware interpolation. As in the voxel-based approach, the intersection points can be computed similarly, i.e. with a line-plane intersection. The resulting area is then weighted with the length of intersection,^22^

(16) $$w_{2}=\frac{\Delta x\Delta y\Delta z}{j_{h}j_{w}e_{x/y}},$$

where Δx/y/z are voxel sizes in each dimension, $j_{w}$ and $j_{h}$ are the width and height of the region enclosed by the four rays in j and $e_{x/y}$ is the normalized direction vector from the detector to the source, either in the XZ -plane ( y ) or in the YZ -plane ( x ). The multiplication of $Area_{f}$ with $w_{2}$ gives the value to be forward projected.

#### Backprojection

The backprojection of BDD is similar to the forward projection, but slightly easier to compute. For the backprojection, we only need integral images of the projection images. Otherwise, the computations are practically identical to forward projection, i.e., the intersection points on i have to be computed along with the area

(17) $$Area_{b}=A_{i}+D_{i}-B_{i}-C_{i}.$$

However, since a separate integral image of the projections is required, the memory cost of the BDD backprojection is larger than that of the voxel-based backprojector. This, however, is alleviated if subsets are used in the reconstruction, as only a subset of the projections needs to be transformed into integral images per subiteration.

The length of intersection weight, however, is different and is given by^22^

(18) $$w_{3}=\frac{\Delta x}{e_{x}^{v}},$$

where Δx is the voxel size in the x dimension and $e_{x}^{v}$ is the normalized direction vector from the voxel to the source in the YZ -plane. As in the forward projection, the XZ -plane is calculated similarly. While some branching is introduced in the backprojection, the effect is negligible as only the weight is different, depending on the plane entered first. The actual value of backprojection is once again computed by multiplying $Area_{b}$ by $w_{3}$ .

For the backprojection, computational efficiency can be improved by reusing previously interpolated values. A single thread can process multiple adjacent voxels, for example, referring to Figure 1, computing the backprojection of the voxel below j with the same thread. In such a case, the interpolated values for iDL and iDR could be reused, and only two additional values would need to be interpolated for the voxel below. This reuse strategy can be extended with additional voxels. In our implementation, using eight voxels per thread seemed to be optimal, but this can easily vary with different hardware. Alternatively, it could be possible to use local/shared memory to compute a group of interpolation values in 2D and reuse these, but this was not implemented. The downside of both methods is that they can increase the error caused by the rotation of the panel in CBCT. As rectangular regions are assumed instead of trapezoidal areas, the more voxels that reuse the previous points, the greater the error in the coordinate to interpolate becomes. In our test cases, the eight voxels worked without noticeable degradation in accuracy.

### Hybrid projectors

In this work, the term *hybrid projector* refers to a projector that uses different projectors for forward and backward projection, specifically a ray-based forward projector and BDD as a backward projector.

An important aspect to consider, especially when using the primal-dual hybrid gradient algorithm (PDHG), is the adjointness of the hybrid methods. PDHG requires the forward and backward projection operations to be adjoint in order to converge.^28^ When using the hybrid projector, exact adjointness is not strictly preserved. While the discrepancy between the left- and right-handed sides of the adjoint condition in (14) is smaller than with ray- and voxel-based combinations without weighting, it is still present. However, this deviation does not pose a practical issue in our setting. Achieving perfect convergence would require thousands of iterations, whereas acceptable reconstruction quality is typically reached in far fewer iterations.

## Iterative reconstruction

Model-based iterative reconstruction is typically based on the minimization of a regularized data fidelity functional

(19) $$\hat{\;f}=\min_{\;f}\frac{1}{2}{\left\|Af-\hat{y}\right\|}_{2}^{2}+\beta H(\;f),$$

where A is the system matrix, f is the image, $\hat{y}$ the (linearized) measurement data, β the regularization parameter, and H(f) the regularization functional. Depending on the regularity of H(f) , different optimization algorithms can be used for the solution of (19).

In this work, we consider test cases reconstructed by means of the least squares estimate with and without regularization ( β=0 in (19)). In both cases, we employ variants of the widely used primal-dual hybrid gradient (PDHG) algorithms.^28,29^ In the case of the least-squares estimate, we employ the PDHG with subsets.^58^ For regularized reconstruction, on the other hand, we use the (stochastic) three-operator variation of PDHG, the Condat-Vũ (CV) algorithm.^59^ The tested projectors should be applicable to any iterative algorithm and have been tested with a variety of algorithms, such as FISTA.^60^ Here, the use of PDHG was also motivated by its requirement of adjointness, meaning that the experiments could reveal if the studied projectors are sufficiently adjoint to be employed with the PDHG algorithms.

### Least squares estimates

In the test cases where we minimize the LS data fidelity (i.e. β=0 in (19)) subject to positivity constraint, we use the dual version of the $L_{2}$ norm PDHG algorithm, as per Algorithm 1,^58^ ${\hat{\;p}}_{k,s}$ is the dual estimate for iteration k and subset s in the projection space, ${\hat{\;f}}_{k,s}$ is the primal (image) estimate, $L_{\mathrm{singular}}$ is the largest singular value of the system matrix $A_{s}$ , σ is the dual step-size value, τ is the primal step-size value and θ is the update step size.

**Table:**
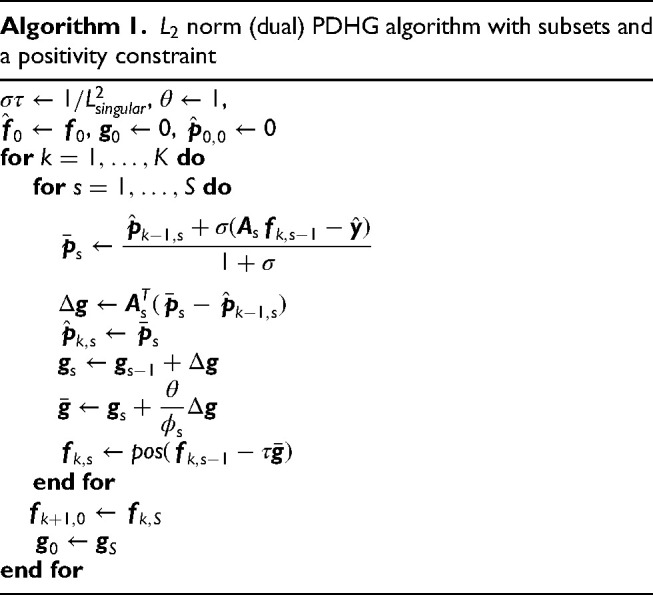


The forward projection ( $A_{s}{\hat{\;f}}_{k,s-1}$ ) and backprojection ( $A_{s}^{T}{\hat{\;p}}_{k,s}$ ) are both computed on-the-fly, regardless of the projector used. This means that matrix $A_{s}$ is never explicitly computed and functions instead as an operator.

### Condat-Vũ algorithm

For the solution of the regularized problem (19), we employ the Condat-Vũ (CV) algorithm, which allows the use of any non-linear convex gradient-based regularization methods. The CV algorithm is described in Algorithm 2, using the same notation as before.

**Table:**
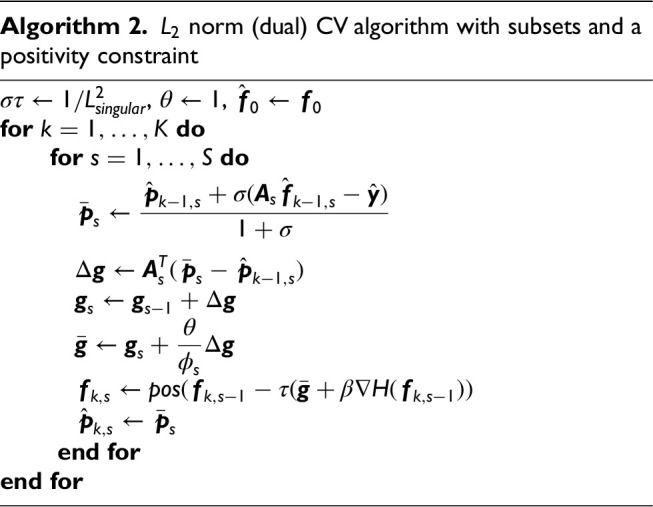


Any linear or non-linear convex gradient-based regularizer could be used with Algorithm 2, but in our case, we use the non-local means (NLM) regularizer.^32^ NLM has previously been used in CT regularization^61–63^ and also as a denoiser.^64–67^ The NLM regularizer is defined as

(20) $$H(f_{j})=\sum_{\iota \in SW_{j}}\left(\omega_{\;j\iota }(f_{j})\phi (f_{j}-f_{\iota })\right),$$

where $SW_{j}$ refers to the *search window*, or neighborhood, which is the region of the selected number of voxels around the voxel j , ϕ is the potential function, in this case $\phi =\Delta f^{2}/2$ . ω is the NLM weight defined as

(21) $$\omega_{\;j\iota }({\hat{\;f}}_{j})=\frac{\exp\;\left(-{\left\|P({\hat{\;f}}_{j})-P({\hat{\;f}}_{\iota })\right\|}_{2,\sigma_{\mathrm{STD}}}^{2}/h_{\mathrm{NLM}}^{2}\right)}{\sum_{\iota \in SW_{j}}\exp\;\left(-{\left\|P({\hat{\;f}}_{j})-P({\hat{\;f}}_{\iota })\right\|}_{2,\sigma_{\mathrm{STD}}}^{2}/h_{\mathrm{NLM}}^{2}\right)},$$

where P refers to the *patch* of the selected number of voxels around the voxels j and ι , $\sigma_{\mathrm{STD}}$ is the standard deviation of the Gaussian weighted Euclidean norm, and $h_{\mathrm{NLM}}$ is a filtering parameter that controls the level of smoothing. The patch window is a separate region centered around either j or ι , and its size, i.e. number of voxels, can be freely selected. The similarity between the patch windows is used to weight the potential function ϕ . Square or cubic regions are usually used for the patch window.

The gradient of (20) is

(22) $$\nabla H(f_{j})=\sum_{\iota \in SW_{j}}\left(\omega_{\;j\iota }(f_{j})(f_{j}-f_{\iota })\right).$$

When computing the gradient in (22), we assume that the weight ω is constant to simplify the differentiation, although the weight is dependent on the image voxel value $f_{j}$ .

## Methods

### Measurement setup

In this study, three different datasets were used: one simulated using an XCAT phantom, and two acquired with real scanners, Planmeca Viso G7 and Planmed XFI. The first dataset consists of simulated scans of the XCAT human phantom,^68^ generated using the open-source MCGPU-software.^69^ The simulated scanner parameters were modeled to match the XFI setup used for the third dataset. This means that the original size of the projection image was chosen to be 1436×1436 , with the simulated detector pixel size of 0.296 mm. The image volume had dimensions of 523×523×523 , with voxel sizes of 0.43 mm in the transaxial direction and 0.48 mm in the axial direction. The distance from the source to the detector was 1086 mm, and from the source to the center of rotation was 600 mm. Three different dose cases were simulated, each with different numbers of photons. The lowest dose case used $5\cdot 10^{9}$ photons per projection, the second doubled this to $1\cdot 10^{10}$ photons per projection, and the third again doubled the previous one to $2\cdot 10^{10}$ photons per projection. In all three cases, 500 projections uniformly distributed over a full 360 ∘ rotation were simulated. This dataset was not used for the FDK evaluations, as the scanner software used for the FDK reconstructions was incompatible with the simulated data.

The second dataset was acquired by scanning a Sedentex phantom with various inserts using the Planmeca Viso G7 head and neck CBCT scanner. A total of 500 projections were taken over 210 ∘ . Each projection image measured 858×858 pixels, with a detector pixel size of 0.278 mm. The reconstruction volume was 568×568×568 with a voxel size of 0.3 mm. The tube voltage was 100 kV, and the current was 100 mAs. The source-to-detector distance was 701.2 mm, and the source-to-rotation-center was 410.7 mm. Due to the design of the scanner, there are some small variations in the distances between different projections.

The third dataset was a scan of an ankle from a human cadaver taken with the Planmed XFI whole-body CBCT scanner. A total of 500 projections were taken over the full 360 ∘ range. The size of the projection image was 1436×1436 with a detector pixel size of 0.296 mm. The size of the original image volume was 523×523×523 , with a voxel size of 0.45 mm. The scan was performed at 100 kV, and 280 mAs. The source-to-detector and source-to-center-of-rotation distances were 1085.9 mm and 601.1 mm, respectively. The cadaver was obtained from the Science Care corporation and donated for science. All approvals for the research use of the cadavers were obtained by the Science Care corporation.

In all cases, only the flat-field correction was applied to the projection data. This correction was performed before the reconstruction. For the real measurements, the correction was applied by the scanner software. For the simulated data, a separate blank scan (without phantom) was used for flat-field correction.

**Figure 2.**
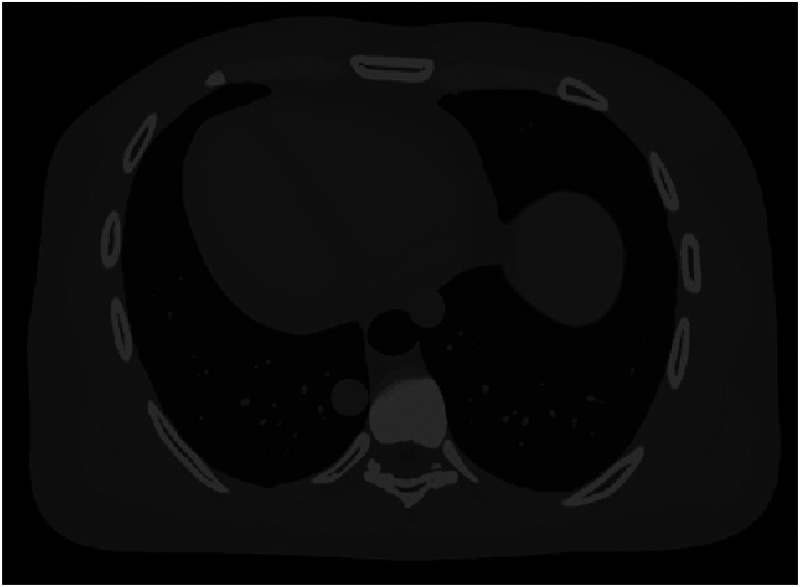
The ground truth image in the simulated case, used in the SSIM and PSNR computations.

To evaluate the reconstruction quality, different metrics were used for each dataset. For the Sedentex phantom, contrast-to-noise ratio (CNR), and modulation transfer function (MTF)^70^ were computed to evaluate the different projectors. The MTF was computed from a square region within a special edge insert, which is shown in the results. For the simulated data, the structural similarity index (SSIM)^71^ and peak signal-to-noise ratio (PSNR)^72^ were computed. Note that due to the output units of eV/cm ${}^{2}$ of the simulations, the units of the reconstructed images do not match the actual attenuation coefficients. Due to this, the simulated data were scaled such that the water equivalent tissue was on average scaled to 0 HU. This scaling was done independently for each reconstruction. The ground truth image was also scaled to HU values similarly. The separate scaling for different dose levels can introduce bias to the fidelity metrics but is necessary due to the mismatch of values between the reconstructions and the ground truth caused by the output units of the simulation. The ground truth image used to compute the SSIM and PSNR values is shown in Figure 2.

### Reconstruction parameters

For the PDHG algorithm, only the primal step-size variable τ was calculated with the power method, while the dual step-size variable σ was fixed at 1. The θ parameter was always set to 1. A total of 20 power method iterations were performed for each dataset to compute τ . All reconstructions were run for 200 iterations using 20 subsets. This number of iterations was chosen because at 200 iterations the least-squares objective values stabilized, and further iterations offered diminishing returns. Although the difference between the 100th and 200th iterations was also rather small, the higher number was chosen to guarantee a more converged image. In practical imaging situations, 100 iterations should be sufficient.

For the fairly standard ray- and voxel-based projector combinations, from here on referred to as the interpolation-based projector, the interpolation length was set to half the voxel length. Hardware-based linear interpolation was used in both the forward and backward projections. Nearest-neighbor interpolation was also tested for backprojection, but linear interpolation produced slightly less noisy images without any computational penalty. Therefore, the results presented in this paper use linear interpolation.

The hybrid projector is very similar to the interpolation-based projector. The forward projection uses the ray-based forward projector as before, but for backprojection uses BDD. The BDD backprojection is computed no differently from the backprojection of the full BDD projector (see section “Backprojection”). Since all the projectors are implemented independently, the hybrid projector does not require a unified memory management strategy: the ray-based forward projector operates in measurement space with detector-centric memory access patterns, while the BDD backprojector works in image space using voxel-centric memory structures. This separation allows each projector to be optimized individually for performance and memory efficiency, without introducing additional overhead.

**Figure 3.**
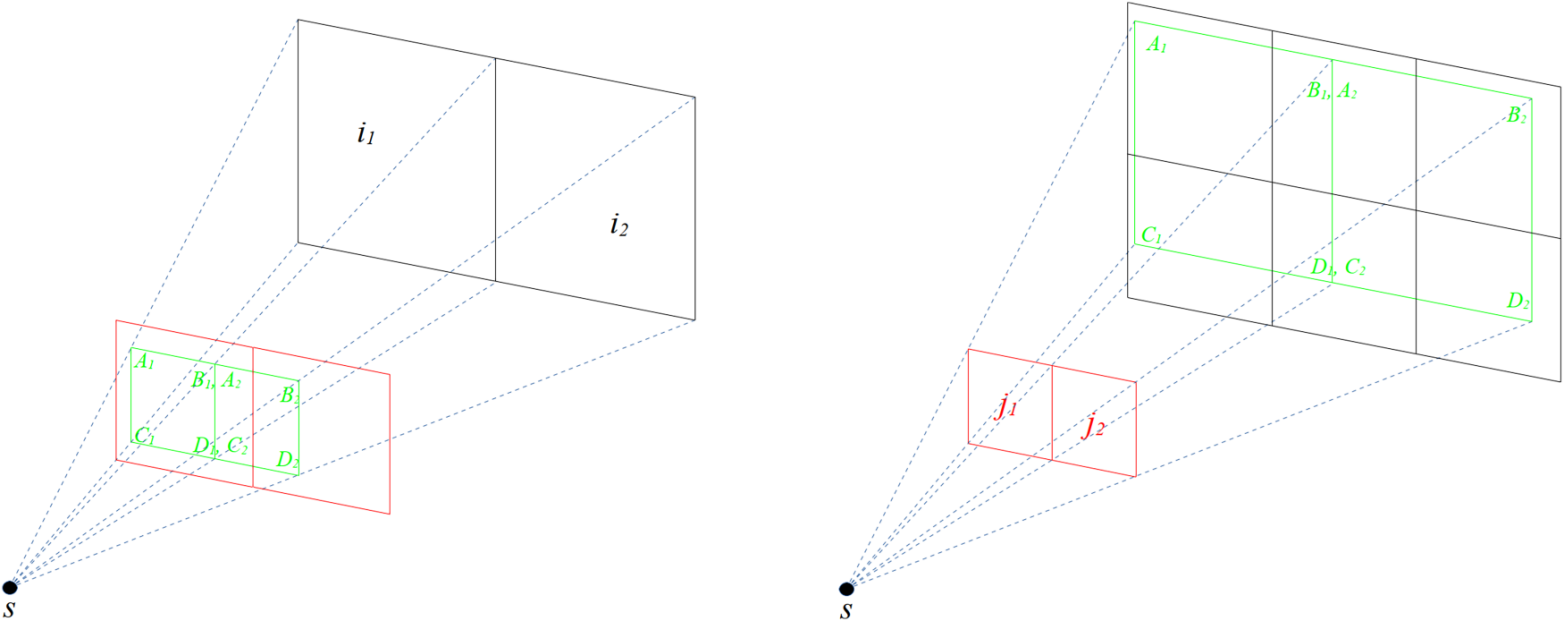
Illustration of the BDD projector in both the forward and backward projections with two adjacent shadows. Black rectangles are individual detector image pixels, green are the BDD shadows, red represent the voxels. The blue dashed lines are the projections from the source to the image or detector that forms the shadow. Left: BDD forward projection, with individual detector pixels marked with the letter j . Right: BDD backprojection, with individual voxels marked with the letter i .

Three different projectors were evaluated: the interpolation-based method, the BDD, and a hybrid projector combining a ray-based forward projector with a BDD backprojector. For details on the functionality of the BDD in both forward and backward projections, see Figure 3. A fourth configuration, using BDD as a forward projector and a voxel-based backprojector, was also tested but yielded significantly worse results and thus omitted from further analysis. For the FDK results, as only the backprojection is used, both the voxel-driven and BDD backprojectors were tested.

Regularized NLM reconstructions were computed for all three data sets. For the Sedentex phantom case, the filter strength $h_{\mathrm{NL}}$ was set to 0.009 and $\sigma_{\mathrm{STD}}$ to 2. The regularization parameter β was set to 200 for the interpolated, 170 for the hybrid, and 160 for the BDD cases. For the cadaver data, the filter strength and $\sigma_{\mathrm{STD}}$ were the same as with the Sedentex data. The regularization parameter β was set to 800 for the interpolated, 400 for the hybrid, and 300 for the BDD cases.

For the simulated data, the parameters were $h_{\mathrm{NL}}=0.015$ and $\sigma_{\mathrm{STD}}=2$ in all three cases. The regularization parameters also varied based on the dose and the projector. For the interpolation-based projector, the regularization parameters were set as $\beta_{1}=4000$ , $\beta_{2}=3000$ , and $\beta_{2}=2500$ from the lowest dose to the highest. Similarly for hybrid: $\beta_{1}=2500$ , $\beta_{2}=2000$ , and $\beta_{2}=1800$ . And for the BDD: $\beta_{1}=2500$ , $\beta_{2}=2000$ , and $\beta_{2}=1800$ . The differences in the regularization parameters for the different methods reflect the varying Lipschitz constants of the projectors and the different noise levels. A larger regularization parameter should be used for noisier data. The filtering parameter $h_{\mathrm{NL}}$ depends on the noise level of the measurement and thus needs to be larger if the data is noisier.

The regularization parameters were selected on the basis of visual assessment. Specifically, each parameter was tuned to reduce noise relative to unregularized reconstruction while preserving structural details and producing a natural-looking texture that would be similar to that of FDK reconstructions. This tuning was performed individually for each projector combination, as one can not expect the parameters to remain consistent despite changes in the projectors. However, since the parameters were chosen based on perceived image quality, this may introduce bias in the comparison of results. All the datasets used a search window size of 5×5×3 and a patch size of 3×3×3 .

For the unregularized cadaver data, we also computed the primal-dual gap values to measure the speed of convergence^29^ between the different projectors. The primal-dual gap value should converge towards zero, but can also be negative before convergence. The primal-dual gap computes the difference between the primal estimate and the dual estimate. Although most LS-based optimization algorithms compute only the estimate equivalent to the primal estimate, the PDHG and its variants also compute the dual estimate. For convergence, it is not sufficient to achieve only zero change in the primal estimate but also in the dual estimate simultaneously. The primal-dual gap was computed with

(23) $$PDg=\frac{1}{2}\|A_{s}{\;f}_{k,s}-{\hat{n}}_{s}{\|}^{2}+\frac{1}{2}\|{\bar{\;p}}_{s}{\|}_{2}^{2}+{\bar{\;p}}_{s}^{T}{\hat{n}}_{s}.$$

The gap values of every subset were then summed together after each iteration, resulting in the same number of gap values as there are iterations.

All non-FDK computations were performed using our open-source software OMEGA,^13^ implemented with OpenCL for GPU acceleration. The computations were run on an Nvidia GeForce 4090 GPU.

For the FDK computations, the Planmeca scanner software PMFDK was used. Reconstructions were performed with and without box filtering, and the results were generated with both voxel-driven and BDD backprojectors.

## Results

The results are divided into three parts. First, we show the comparisons of the three different projectors without regularization using the PDHG algorithm. Second, we evaluate the same projectors with NLM regularization using the CV algorithm. Finally, we present FDK reconstructions computed with both BDD and voxel-driven backprojectors.

The unregularized PDHG reconstructions were performed on the simulated dataset, the Sedentex phantom, and the cadaver ankle. NLM regularization was applied to the simulated and cadaver datasets, representing different dose scenarios, respectively. FDK reconstructions were evaluated using the cadaver ankle and Sedentex datasets.

The unregularized reconstructions highlight the differences between the projectors in an unmodified setting where the image quality is only affected by the choice of the projector and the number of iterations. Although varying the number of iterations can act as a type of regularization for all projectors, in this case, a sufficiently high number of iterations was used to ensure convergence, allowing for a fair comparison of the projector models themselves.

The regularized reconstructions present a more realistic practical use case, as model-based iterative reconstruction methods almost always include regularization. The regularized reconstructions show the differences between the projectors in the presence of the denoising NLM regularizer, which removes the potential denoising effect of the projectors and shows their potential, or lack of, improvement in image quality due to the different modeling of the geometry. The downside of using regularized reconstructions is that some bias is introduced through the tuning of regularization parameters.

The performance of the BDD projector is closely related to the size of the center shadow, i.e. the side length of the region i in Figure 1 computed from a voxel in the middle of the image volume. Since the voxels are cubic, the region is square. Larger shadow sizes amplify the benefits of using the BDD projector. Therefore, we also report the length of the central shadow region for each dataset, i.e. the size of the shadow in the backprojection when viewed from the volume’s center voxel.

### Comparisons between different projectors - least-squares reconstructions using PDHG

PDHG, implemented with the Algorithm 1 was used here for all three datasets.

**Figure 4.**
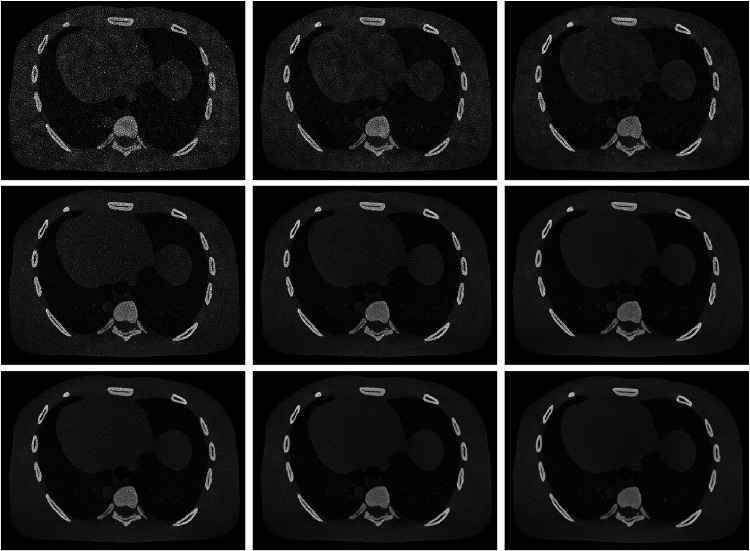
A transverse slice from the simulated MCGPU dataset. From top to bottom: Interpolation, hybrid, and BDD projectors. From left to right: From lowest to highest dose. The color scale is [−1000,2000] HU.

Figure 4 shows a close-up region of transverse slices from the reconstructed MCGPU XCAT-phantom data. The color scale is set to [−1000,2000] HU. The top row shows the interpolation projector reconstructions, the middle hybrid, and the bottom BDD. While the left column shows the lowest dose of $5\cdot 10^{9}$ photons, the center $1\cdot 10^{10}$ photons, and the right $2\cdot 10^{10}$ photons.

Figure 4 shows that the BDD projector produces noticeably less noisy images compared to the interpolation-based method. The hybrid projector appears to be right in the middle in terms of image quality. The BDD allows some details to be seen, such as the small details of the lungs, which are much more difficult to see from the reconstruction with the interpolation-based projector. The differences between the methods decrease as the dose increases, with the greatest differences seen with the lowest dose. Especially the hybrid and BDD become very similar in the highest dose case. The center shadow length in this case is 0.720 mm. The computation times per epoch, or full iteration (with all subsets) are shown in Table 1. The difference in computation times between the interpolation-based method and the hybrid projector is only about 0.4 s, while the BDD is about twice as slow.

**Table 1.** Computation times for the different projectors for one epoch when using simulated data.

Projector	Computation time (s)
Interpolation	2.39
BDD backprojector (hybrid)	2.83
BDD	4.98

**Figure 5.**
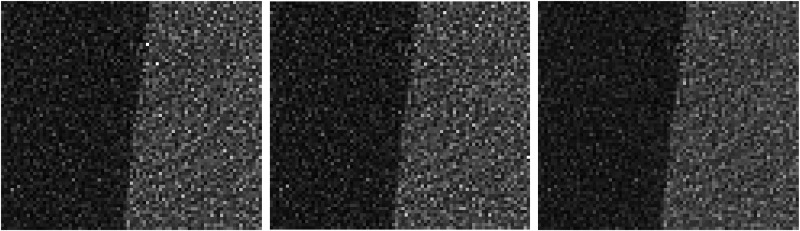
Close-up region of a transverse slice of the Sedentex phantom dataset used in the MTF calculations. Left: Interpolation-based projector. Middle: Hybrid with ray-based forward projector and BDD backprojector. Right: BDD projector. The color scale is [−750,750] HU.

Table 2 shows the SSIM and PSNR values computed from the ground truth image, shown in Figure 2. The SSIM and PSNR values from the table are in close agreement with the visual results; the BDD projector achieves the best results, followed by hybrid, with the interpolation-based method performing the worst of the three. From both SSIM and PSNR values, it is also possible to see that the differences between the methods diminish as the dose increases. Figure 5 shows a close-up region of a transverse slice of the Sedentex-phantom. The color scale is [−750,750] HU. From Figure 5, we can see that the BDD method leads to a slightly less noisy image compared to the interpolation-based one. The hybrid shows similarities with both the interpolation and fully BDD approaches but resembles the interpolation-based result more. The length of the shadow for the Sedentex phantom data is 0.459 mm. The computation times per one full iteration (epoch) are shown in Table 3. These results are consistent with the simulated case, although the relative overhead of the BDD is slightly reduced.

**Figure 6.**
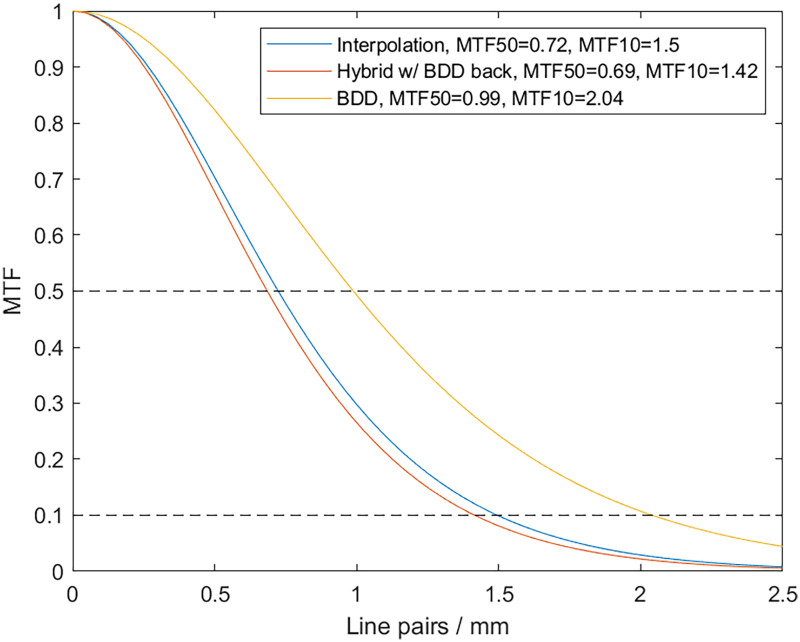
MTF values for each projector, including the MTF at 50% and MTF at 10% values. Computed from the edge in Figure 5.

MTF values are presented in Figure 6, derived from the edge shown in Figure 5. The figure includes both the MTF50 and MTF10 values, which provide a quantitative measure of spatial resolution.

**Table 2.** SSIM and PSNR values for the simulated data for all three projectors.

No. photons	$5\cdot 10^{9}$		$1\cdot 10^{10}$		$2\cdot 10^{10}$	
Projector	SSIM	PSNR	SSIM	PSNR	SSIM	PSNR
Interpolation	0.95896	42.951	0.97525	44.830	0.98572	46.535
BDD backprojector (hybrid)	0.98303	45.975	0.99009	47.397	0.99367	48.337
BDD	0.99200	47.802	0.99484	48.630	0.99621	49.117

**Table 3.** Computation times for the different projectors for one epoch when using the sedentex phantom data.

Projector	Computation time (s)
Interpolation	1.66
BDD backprojector (hybrid)	2.17
BDD	3.29

As can be seen in Figure 6, the BDD achieves the highest MTF values, indicating superior spatial resolution. The hybrid and interpolation-based projectors are very close to each other, with the hybrid having slightly worse MTF values. These numbers align with the visual inspection of Figure 5, where the BDD gives a less noisy image, while maintaining edge sharpness.

**Figure 7.**
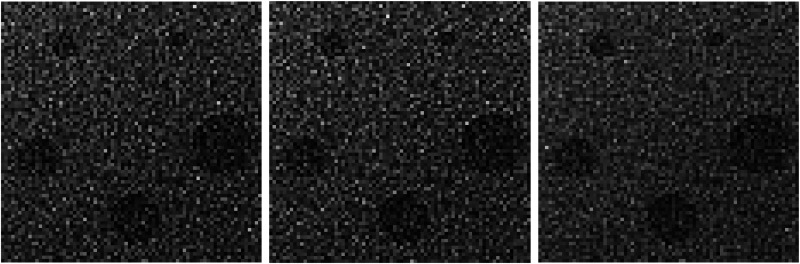
Close-up region of a transverse slice of the Sedentex phantom dataset used in the CNR calculations. Left: Interpolation-based projector. Middle: Hybrid with ray-based forward projector and BDD backprojector. Right: BDD projector. The color scale is [−500,500] HU.

Figure 7 shows a close-up region of a transverse slice of the Sedentex phantom dataset. The color scale is [−500,500] HU. This region was used to compute the CNR values shown in Table 4. The CNR was computed from the three largest spheres, with the background values taken from adjacent regions of similar size.

**Table 4.** CNR values for the sedentex phantom dataset for the region shown in Figure 7.

Projector	CNR
Interpolation	0.5443
BDD backprojector (hybrid)	0.5680
BDD	0.6600

Among the three methods, the BDD backprojectpr yields the highest CNR values, followed by the hybrid approach. However, the difference between the hybrid and the interpolation-based projector is small, whereas the improvement offered by the BDD projector is substantial and the difference when compared to interpolation is visually apparent in Figure 7.

**Figure 8.**
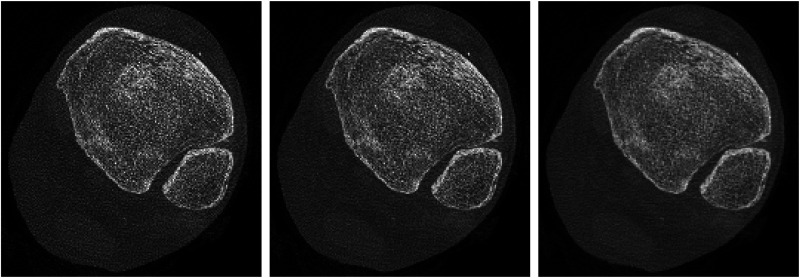
Close-up region of a transverse slice from the cadaver ankle dataset. Left: Interpolation-based projector. Middle: Hybrid with ray-based forward projector and BDD backprojector. Right: BDD projector. The color scale is [−1000,1800] HU.

Figure 8 shows a close-up region of a transverse slice from the cadaver ankle data. The color scale is [−1000,1800] HU.

As with the Sedentex phantom dataset, the cadaver ankle exhibits similar trends, but with more pronounced differences between the methods. The interpolation-based projector is again the noisiest, and the BDD produces the cleanest result. The hybrid projector performs closer to the BDD in terms of noise suppression, more so than with the Sedentex data. The BDD clearly shows better contrast with the soft tissue, as the different soft tissue regions under the bone can be seen more clearly. For the bone region, the interpolation-based projector provides a very sharp-looking image, making the texture appear overly enhanced and unrealistic due to the elevated noise levels. The BDD, although arguably blurrier, offers a more natural-looking texture. The hybrid projector strikes a balance between the two, offering a good compromise not only computationally but also in terms of image quality.

**Figure 9.**
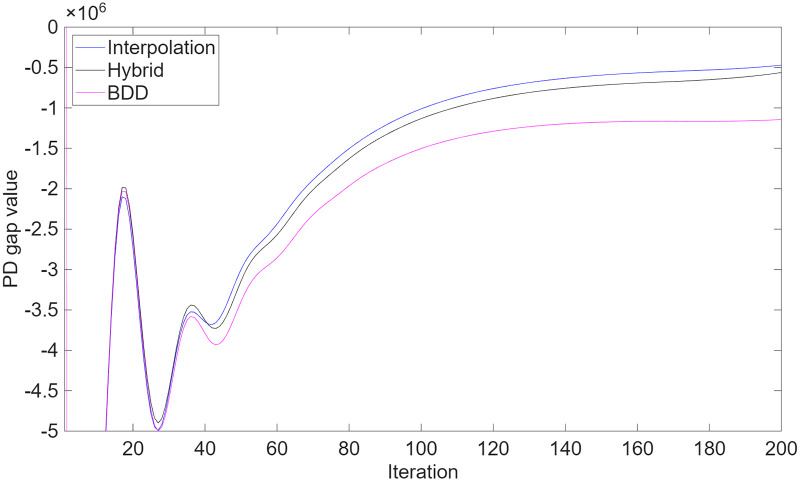
The primal-dual gap values, computed with (23), for the XFI cadaver ankle data.

The primal-dual gap values, calculated with (23), for the three projectors are shown in Figure 9.

Based on Figure 9, the BDD projector has the slowest convergence rate, while the hybrid and interpolation-based projectors are very close to each other. If the interpolation-based or hybrid projector reconstructions are viewed at the iteration number where the gap value is closest to the BDD, the image quality still favors the BDD. However, both the hybrid and the interpolation-based reconstructions have slightly less noise at that iteration.

The center shadow length is 0.876 mm in this case. The computation times per one full iteration (epoch) are shown in Table 5. As before, while the difference between the interpolation-based and the hybrid projector is not that significant, the BDD remains the most computationally intensive.

**Table 5.** Computation times for the different projectors for one epoch when using the XFI ankle cadaver data.

Projector	Computation time (s)
Interpolation	3.22
BDD backprojector (hybrid)	3.97
BDD	5.31

### Comparisons between different projectors using CV with NLM regularization

This section presents results for the optimization problem (19) using the NLM regularization computed with the CV Algorithm 2. The evaluation focuses on the same datasets as the non-regularized cases.

We want to remark that selecting a regularization parameter that ensures an identical level of regularization is a challenge. As a result, the comparisons are not as straightforward as those based on the pure LS estimates.

**Figure 10.**
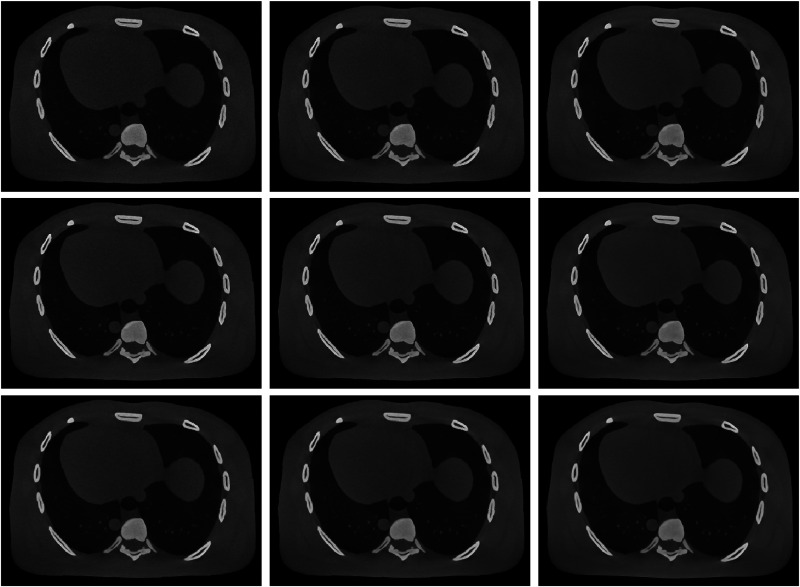
Close-up region of a transverse slice from the reconstructed MCGPU dataset with NLM regularization. From top to bottom: Interpolation, hybrid, and BDD projectors. From left to right: From lowest to highest dose. The color scale is [−1000,2000] HU.

Figure 10 shows a close-up region of transverse slices from the NLM regularized reconstruction of MCGPU XCAT-phantom data. The color scale is set to [−1000,2000] HU. The top row shows interpolation projector reconstructions, the middle hybrid, and the bottom BDD. While the left column shows the lowest dose of $5\cdot 10^{9}$ photons, the center $1\cdot 10^{10}$ photons, and the right $2\cdot 10^{10}$ photons.

The interpolation-based projector exhibits visibly lower performance in preserving anatomical details, particularly in the lung region, compared to the hybrid and BDD projectors. The details of the lung have better contrast with both the hybrid and the BDD when compared with the interpolation-based projector, but otherwise the differences are small. Furthermore, the differences between the methods diminish with increasing dose. In general, the hybrid and BDD reconstructions appear nearly identical, making it difficult to distinguish between them visually. The benefits of the BDD, especially the backprojector part of the BDD, appear to be emphasized when the dose is decreased or the number of projections is reduced. This becomes evident when the simulated results are compared with the measured cadaver results shown below.

Table 6 lists the SSIM and PSNR values computed from the ground truth image, shown in Figure 2, for the NLM regularized reconstructions.

**Table 6.** SSIM and PSNR values for the simulated data for all three projectors in the regularized NLM reconstructions.

No. photons	$5\cdot 10^{9}$		$1\cdot 10^{10}$		$2\cdot 10^{10}$	
Projector	SSIM	PSNR	SSIM	PSNR	SSIM	PSNR
Interpolation	0.99735	49.443	0.99743	49.546	0.99753	49.644
BDD backprojector (hybrid)	0.99743	49.449	0.99750	49.553	0.99757	49.649
BDD	0.99761	49.533	0.99768	49.639	0.99775	49.733

The SSIM values from Table 6 indicate that both the BDD and hybrid projectors outperform the interpolation-based projector. However, comparing the values of Table 6 with that of Table 2, we can see that the differences between the three projectors diminish greatly when regularization is used. The SSIM and PSNR values also do not vary significantly with varying dose levels. Although visually differences can be seen especially when comparing the interpolation-based projector with the hybrid and BDD, the SSIM and PSNR values show only very small variations.

**Figure 11.**
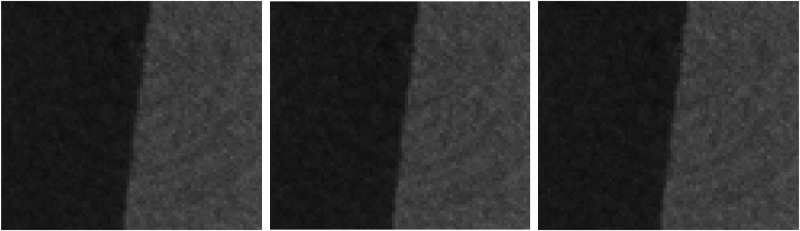
Close-up region of a transverse slice of the Sedentex phantom dataset for the regularized case, used in the MTF calculations. Left: Interpolation-based projector. Middle: Hybrid with ray-based forward projector and BDD backprojector. Right: BDD projector. The color scale is [−750,750] HU.

**Figure 12.**
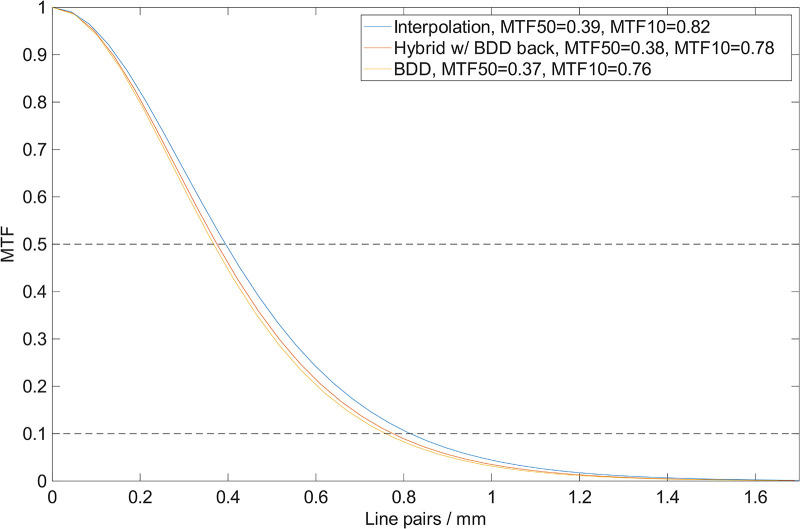
MTF values for each projector for the regularized case, including the MTF at 50% and MTF at 10% values. Computed from the edge in Figure 11.

Figure 11 shows a close-up region of a transverse slice of the Sedentex-phantom for the regularized case. The color scale is [−750,750] HU. Unlike in the unregularized case, it is difficult to see any differences between the three methods. The same applies to the MTF values, shown in Figure 12, where there are only small differences between the methods.

**Figure 13.**
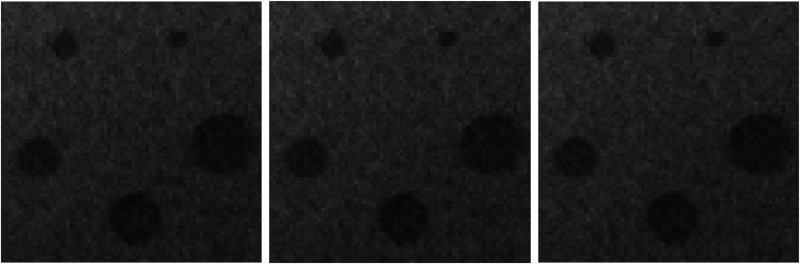
Close-up region of a transverse slice of the Sedentex phantom dataset for the regularized case used in the CNR calculations. Left: Interpolation-based projector. Middle: Hybrid with ray-based forward projector and BDD backprojector. Right: BDD projector. The color scale is [−500,500] HU.

Figure 13 shows a close-up region of a transverse slice of the Sedentex phantom dataset in regularized case and also the slice used for the calcuation of the CNR values. The color scale is [−500,500] HU. The CNR values are shown in Table 7. The CNR was computed from the three largest spheres, with the background values taken from adjacent regions of similar size. As with the MTF values, the CNR values also exhibit only minor differences between the three projectors.

**Table 7.** CNR values of the regularized case for the sedentex phantom dataset for the region shown in figure 13.

Projector	CNR
Interpolation	2.1284
BDD backprojector (hybrid)	2.1237
BDD	2.1294

**Figure 14.**
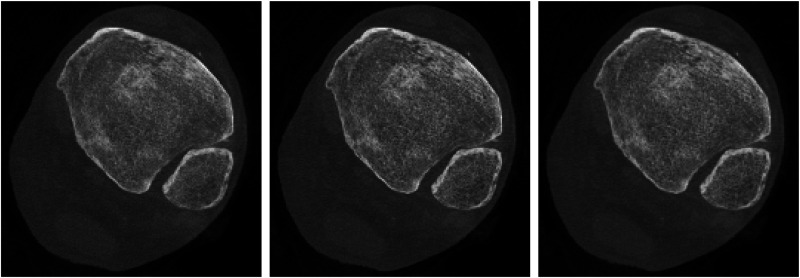
Close-up region of a transverse slice of the cadaver ankle dataset with NLM regularization. Left: Interpolation-based projector. Center: Hybrid with ray-based forward projector and BDD backprojector. Right: BDD projector. The color scale is [−1000,1800] HU.

In Figure 14, we present a close-up region of the cadaver ankle dataset similar to the one in Figure 8. The color scale is [−1000,1800] HU.

When comparing the Figures 8 and 14, it becomes evident that the differences between the projectors become negligible. Although minor variations can be observed upon close inspection, the overall image quality is comparable across all methods. This suggests that the benefits of the BDD and hybrid projectors are not as apparent. Given their higher computational cost, the interpolation-based projector may be the most practical choice for standard-dose imaging scenarios.

### FDK reconstruction with BDD

**Figure 15.**
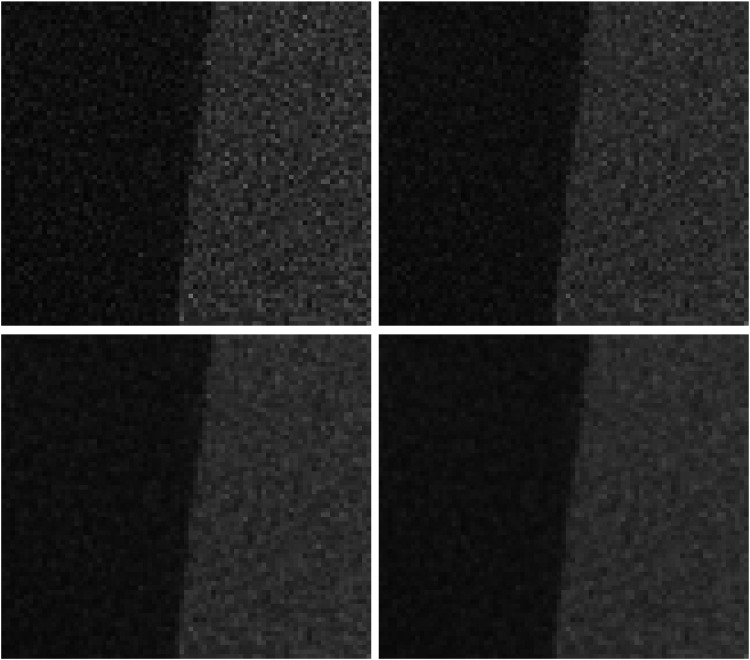
Close-up region of a transverse slice of the Sedentex phantom dataset used in the MTF calculations. Top left: Voxel-driven, unfiltered. Top right: BDD, unfiltered. Bottom left: Voxel-driven, filtered. Bottom right: BDD, filtered. The color scale is [−750,750] HU.

Figure 15 presents a close-up region of a transverse slice of the Sedentex phantom reconstructed with FDK with optional filtering. The color scale is [−750,750] HU. Both unfiltered and filtered cases of voxel-driven and BDD backprojectors are shown.

The difference between the unfiltered cases in Figure 15 is larger than the difference between the filtered ones. However, the unfiltered BDD has a certain similarity to the filtered voxel-driven. In all cases though, the BDD gives smoother results.

**Figure 16.**
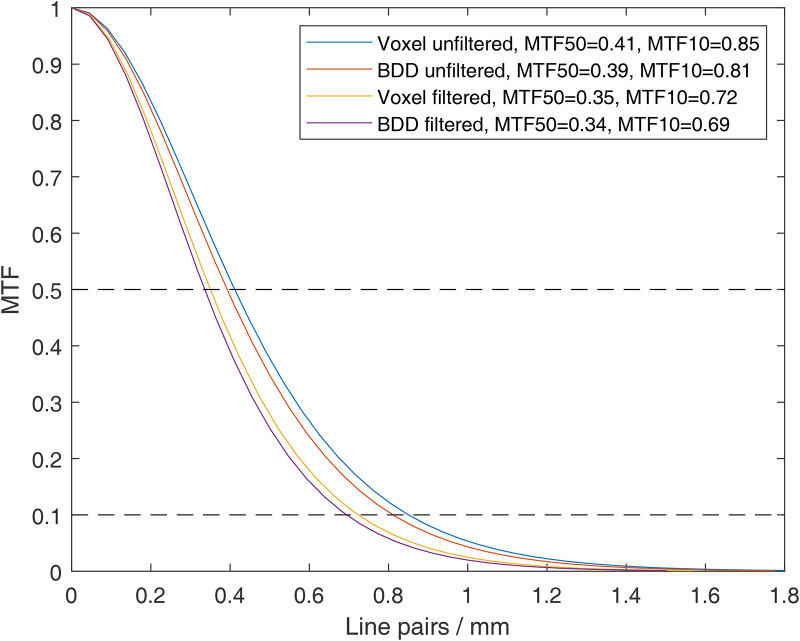
MTF values for each projector, including the MTF at 50% and MTF at 10% values. Computed from the edge in Figure 15.

MTF values (both the MTF50 and MTF10 ones) are displayed in Figure 16, computed from the edge insert which can be seen in Figure 15.

The MTF values show only minor differences among the four methods. The smoother images correspond to lower MTF values. This leads to the unfiltered voxel-driven achieving the largest MTF values, followed closely by the unfiltered BDD. The difference between the filtered versions is even less than with the unfiltered ones, which indicates that the filtering mitigates the inherent differences between the two projectors.

**Figure 17.**
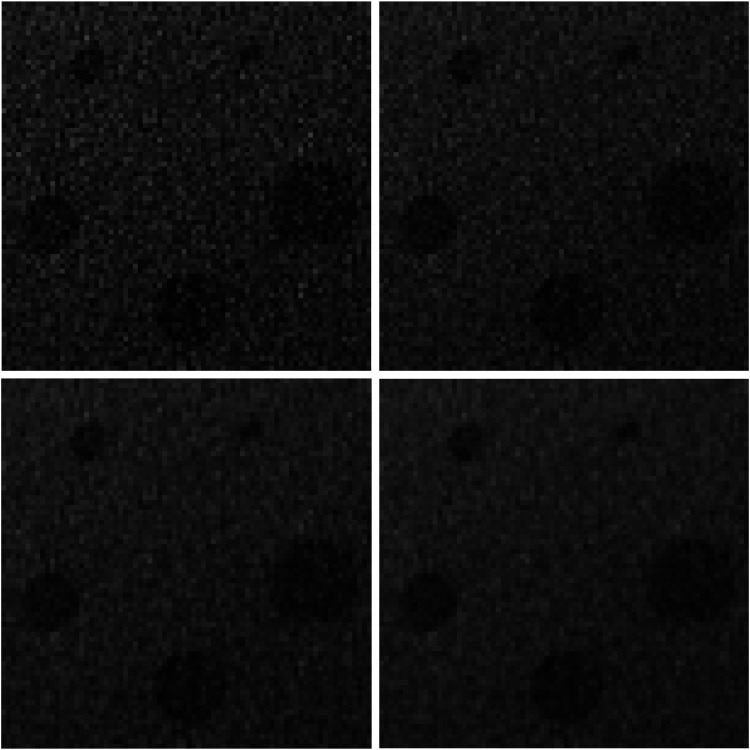
Close-up region of a transverse slice of the Sedentex phantom dataset used in the CNR calculations. Top left: Voxel-driven, unfiltered. Top right: BDD, unfiltered. Bottom left: Voxel-driven, filtered. Bottom right: BDD, filtered. The color scale is [−500,500] HU.

Figure 17 displays the close-up region of a transverse slice of the Sedentex phantom data used for CNR analysis, with the results summarized in Table 8. The color scale is [−500,500] HU. As before, the CNR was computed based on the three largest circles, with the background values computed from similarly sized regions adjacent to the circles.

**Table 8.** CNR values for the sedentex dataset for the region shown in figure 17. FDK reconstructions.

Backprojector	CNR
Voxel-driven, unfiltered	0.9525
BDD, unfiltered	1.1954
Voxel-driven, filtered	1.4569
BDD, filtered	1.6757

When comparing Figure 17 and the CNR values, the results are in good agreement with each other. From both the table and the figure, it is clear that the BDD backprojector performs better than the voxel-driven backprojector. Based on the CNR values, the voxel-driven backprojector benefits more from the additional filtering compared to the BDD. This is not surprising, as the unfiltered BDD already has similar CNR values to the filtered voxel-driven approach.

**Figure 18.**
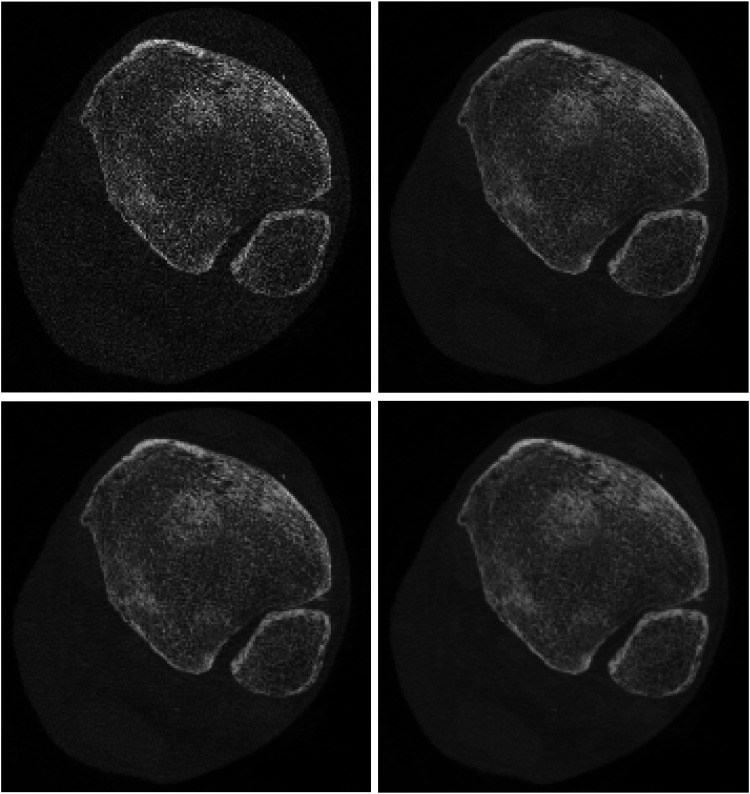
Example FDK reconstruction with the cadaver ankle dataset, reconstructed by the XFI scanner software. Top left: Voxel-driven, unfiltered. Top right: BDD, unfiltered. Bottom left: Voxel-driven, filtered. Bottom right: BDD, filtered. The color scale is [−1000,1800] HU.

In Figure 18, we show an example reconstruction of the cadaver ankle data with FDK. As with the Sedentex phantom, both filtered and unfiltered versions are shown for each backprojector. The slice is the same as in Figure 7, and the color scale is the same [−1000,1800] HU.

The results of Figure 18 show larger differences between the backprojectors than with the Sedentex dataset, likely due to the larger shadow size. In this case, the difference between the unfiltered BDD and filtered voxel-driven methods is very small. The unfiltered voxel-driven backprojector produces a very low-quality reconstruction, while the unfiltered BDD offers much better quality. The image is less noisy, and the contrast is significantly better than the unfiltered voxel-driven reconstruction. Visually, the contrast of the unfiltered BDD reconstruction appears to be slightly better than the filtered voxel-driven one. The filtered BDD offers the smoothest image and most uniform texture, allowing soft tissue regions to be more easily distinguishable.

The results indicate that the BDD-based FDK is less dependent on filtering. However, as with the iterative reconstructions, the smoothing effect of the BDD is dependent on the scanner geometry, stronger with the XFI ankle data than with the Viso Sedentex data. The BDD-based FDK could allow for removal of filtering with certain scanners or enhance the image quality with an optimized combination of filtering when compared with the voxel-based backprojector.

## Discussion

In this paper, we compared the branchless distance-driven (BDD) projector with an interpolation-based projector for CBCT using a primal-dual algorithm. Initially, we showcased the potential of a hybrid projector, using the BDD as a backprojector with a ray-based forward projector, both in terms of image quality and computational efficiency. Reconstructions were evaluated with and without regularization in a model-based iterative reconstruction framework. Furthermore, we explored the potential of the BDD as a backprojector within the FDK reconstruction pipeline.

Based on the results, the BDD often yielded superior image quality compared to an interpolation-based approach. The improvement was particularly evident in the least squares reconstructions at normal-dose level, but when regularization was applied to the same data, the difference between projectors became smaller but BDD still provided slightly better image quality. A similar trend was observed for the hybrid projector when compared with the interpolation-based approach.

In low-dose scenarios, the BDD projector consistently produced better image quality, both in unregularized LS and even for NLM regularized reconstructions. The improvement yielded with the BDD appears to depend on both the dose and the size of the shadow. The hybrid projector performed almost as well as the BDD while offering significantly faster computational times. For instance, in the cadaver ankle dataset, the hybrid projector was twice as fast as the BDD and only about 15% slower than the interpolation-based projector. This makes the hybrid projector a compelling alternative to BDD, when computation time is critical.

Quantitative analysis of the results in this work is based on the fidelity metrics CNR, MTF, SSIM, and PSNR. However, in an actual clinical study, these metrics may not directly correlate with the diagnostic task and radiologist’s view on the images, so care has to be taken in interpretation of medical images based on these metrics. For instance, in some cases a reconstruction exhibiting a smaller SSIM might be clinically more useful than one with a higher value. Thus, the final evaluation should always be based on the clinical requirements for image quality in a particular application.

The BDD projector can be considered to have a geometry-specific regularizing effect. It has a similar smoothing and denoising effect to traditional regularizers, but its effectiveness is influenced by the geometry of the scanning setup, particularly the shadow size in both forward and backward projections. When the shadow size is small relative to the detector pixel or voxel size, the differences between BDD and conventional methods diminish. In CBCT applications, it is common for scanning setups to vary significantly depending on the scanner type used and the examination. This means that the BDD is not automatically an optimal choice when it comes to image quality. The hybrid projector, with BDD as the backprojector, retains the same properties as BDD in the backprojection but is not dependent on the shadow size in the forward projection. Another advantage of using only the BDD backprojector, is the memory usage, as BDD forward projector requires two full image volumes to be stored. This can require significantly more memory than a ray-based approach if high-dimensional image volumes are reconstructed. The BDD backprojector does not have a similar shortcoming as it only requires one integral image, and with the use of subsets, the size of the integral image can be reduced.

Based on this work, the BDD and the hybrid projectors can be a good choice when the shadow size is larger than the detector pixel size. The larger the shadow, the greater the benefit. However, the effect is smaller if regular dose levels and regularization techniques are applied. Since the effect on image quality depends on both the shadow size and dose, it is not possible to provide clear guidelines on when the BDD or the hybrid method would be the most optimal. The shadow size is also affected by several variables, such as the detector pixel size, the voxel size, and the relative positions of the source, object, and detector. For forward projection, the BDD gives greater benefit if the voxel size is small relative to the detector pixel size, while for backprojection the opposite is true.

As we demonstrate here, the BDD backprojector is a viable alternative in FDK reconstructions. The benefits of the BDD in FDK cases are again geometry-dependent. In the case of the XFI dataset, which featured a larger shadow size, the unfiltered BDD reconstruction closely matched the quality of the filtered voxel-driven one. In contrast, the Sedentex measurement (with smaller shadow size) showed smaller differences, though the trend is the same for both datasets; the BDD offers smoother and less noisy reconstructions than the voxel-driven backprojector. Both the CNR and MTF values measured with the Sedentex phantom favor the BDD reconstructions. Since FDK involves computing only one backprojection, the computational cost differences between the backprojectors become negligible.

The BDD forward and backward projections rely on the integral image to compute the area. This computation assumes that the projected area is rectangular. In most CBCT scanners, the panel is slightly rotated such that the area is not perfectly rectangular. However, this rotation is typically small enough that it has no noticeable effect on the reconstruction quality. If the imaging setup involves a more complex geometrical setup with a significantly rotate panel, the rectangular assumption will no longer hold, and the accuracy of the BDD projector may degrade. Therefor, BDD-based methods are best suited for geometries where the projected area is at least approximately rectangular.

All projectors and methods presented in this paper, except for the FDK implementation, have been implemented as part of the v2.0 release of the open-source multi-dimensional tomography reconstruction software (OMEGA)^13^ available on GitHub: https://github.com/villekf/OMEGA/. The implementation supports MATLAB, GNU Octave, and Python.

Based on the results presented in this paper, the following conclusions are made. In least squares reconstructions, the BDD leads to better image quality than the interpolation-based or hybrid methods, as long as the shadow size, taken from a voxel in the center of the image volume, is larger than a single detector pixel. In regularized reconstructions, the advantage of BDD diminishes in densely sampled low noise (normal CBCT dose level) situations. For low-dose CBCT, the BDD projector leads to improved reconstruction quality both with and without regularization, suggesting that BDD could be especially beneficial in the development of low-dose imaging applications. The hybrid projector, which uses BDD only for backprojection, offers improved image quality and computational performance comparable to that of the interpolation-based method, provided that the full BDD projector improves the image given the scanning setup. Lastly, for FDK reconstructions, the BDD is a viable alternative for backprojection, producing very good noise removal, when the shadow size is sufficiently large.

## References

[1] Hanke R Fuchs T Uhlmann N . X-ray based methods for non-destructive testing and material characterization. Nuclear Inst Methods Phys Res Sec A: Acceler Spect Detect Assoc Equipment 2008; 591: 14–18.

[2] Evans L Margetts L Casalegno V , et al. Transient thermal finite element analysis of CFC–cu ITER monoblock using X-ray tomography data. Fusion Eng Design 2015; 100: 100–111.

[3] Dambrogio J Ghassaei A Smith DS , et al. Unlocking history through automated virtual unfolding of sealed documents imaged by X-ray microtomography. Nat Commun 2021; 12: 1184. 33654094 10.1038/s41467-021-21326-w PMC7925573

[4] Seales WB Parker CS Segal M , et al. From damage to discovery via virtual unwrapping: Reading the scroll from en-gedi. Sci Adv 2016; 2: e1601247. 10.1126/sciadv.1601247 PMC5031465 27679821

[5] Tonai S Kubo Y Tsang MY , et al. A new method for quality control of geological cores by X-ray computed tomography: Application in IODP expedition 370. Front Earth Sci 2019; 7. DOI: 10.3389/feart.2019.00117.

[6] Castañón DA Babaheidarian P . Joint reconstruction and material classification in spectral CT. In: Ashok A, Neifeld MA, Gehm ME et al. (eds) *Anomaly detection and imaging with X-Rays (ADIX) III*. SPIE. DOI: 10.1117/12.2309663.

[7] Megherbi N Flitton GT Breckon TP . A classifier based approach for the detection of potential threats in CT based baggage screening. In: *2010 IEEE International conference on image processing*. IEEE. DOI: 10.1109/icip.2010.5653676.

[8] Wang G . X-ray micro-CT with a displaced detector array. Med Phys 2002; 29: 1634–1636. 12148746 10.1118/1.1489043

[9] De Man B Basu S . Distance-driven projection and backprojection. In: *2002 IEEE Nuclear science symposium conference record*, Vol. 3, pp.1477–1480. IEEE. DOI: 10.1109/NSSMIC.2002.1239600.

[10] Jia X Lou Y Lewis J , et al. GPU-based fast low-dose cone beam CT reconstruction via total variation. J X-Ray Sci Technol 2011; 19: 139–154. 10.3233/XST-2011-0283 21606579

[11] Park HG Shin YG Lee H . A fully GPU-based ray-driven backprojector via a ray-culling scheme with voxel-level parallelization for cone-beam CT reconstruction. Technol Cancer Res Treat 2015; 14: 709–720. 24750005 10.7785/tcrt.2012.500429

[12] Long Y Fessler JA Balter JM . 3D forward and back-projection for X-ray CT using separable footprints. IEEE Trans Med Imag 2010; 29: 1839–1850. 10.1109/TMI.2010.2050898 PMC2993760 20529732

[13] Wettenhovi VV Vauhkonen M Kolehmainen V . OMEGA—open-source emission tomography software. Phys Med Biol 2021; 66: 065010. 33588401 10.1088/1361-6560/abe65f

[14] Zeng G Gullberg G . A ray-driven backprojector for backprojection filtering and filtered backprojection algorithms. In: *1993 IEEE conference record nuclear science symposium and medical imaging conference*. NSSMIC-93, 1993, IEEE. DOI: 10.1109/nssmic.1993.701833.

[15] Zwicker M Pfister H van Baar J , et al. Ewa splatting. IEEE Trans Visualiz Comput Graphics 2002; 8: 223–238.

[16] Fessler JA . Penalized weighted least-squares image reconstruction for positron emission tomography. IEEE Trans Med Imaging 1994; 13: 290–300. 18218505 10.1109/42.293921

[17] Liu R He L Luo Y , et al. Singular value decomposition-based 2D image reconstruction for computed tomography. J X-Ray Sci Technol 2017; 25: 113–134. 10.3233/XST-16173 27834789

[18] Lougovski A Hofheinz F Maus J , et al. A volume of intersection approach for on-the-fly system matrix calculation in 3D PET image reconstruction. Phys Med Biol 2014; 59: 561–577. 24434600 10.1088/0031-9155/59/3/561

[19] Sutherland IE Hodgman GW . Reentrant polygon clipping. Commun ACM 1974; 17: 32–42.

[20] Yu H Wang G . Finite detector based projection model for high spatial resolution. J X-Ray Sci Technol 2012; 20: 229–238. 10.3233/XST-2012-0331 PMC3807242 22635177

[21] Man BD Basu S . Distance-driven projection and backprojection in three dimensions. Phys Med Biol 2004; 49: 2463–2475. 15248590 10.1088/0031-9155/49/11/024

[22] Liu R Fu L De Man B , et al. GPU-based branchless distance-driven projection and backprojection. IEEE Trans Comput Imag 2017; 3: 617–632. 10.1109/TCI.2017.2675705 PMC5761753 29333480

[23] Schlifske D Medeiros H . A fast GPU-based approach to branchless distance-driven projection and back-projection in cone beam CT. In: Kontos D, Flohr TG and Lo JY (eds) *Medical imaging 2016: Physics of medical imaging*. SPIE. DOI: 10.1117/12.2216628.

[24] Mitra A Politte DG Whiting BR , et al. Multi-GPU acceleration of branchless distance driven projection and backprojection for clinical helical CT. J Imag Sci Technol 2017; 61: 010405. 10.2352/J.ImagingSci.Technol.2017.61.1.010405 PMC5448423 28572719

[25] Xie X McGaffin MG Long Y , et al. Accelerating separable footprint (SF) forward and back projection on GPU. In: Flohr TG, Lo JY and Gilat Schmidt T (eds) *SPIE Proceedings*, 2017. SPIE. DOI: 10.1117/12.2252010.

[26] Basu S De Man B . Branchless distance driven projection and backprojection. In: Bouman CA, Miller EL and Pollak I (eds) *SPIE Proceedings*, 2006. SPIE. DOI: 10.1117/12.659893.

[27] Feldkamp LA Davis LC Kress JW . Practical cone-beam algorithm. J Opt Soc Am A 1984; 1: 612–619.

[28] Chambolle A Pock T . A first-order primal-dual algorithm for convex problems with applications to imaging. J Math Imag Vision 2011; 40: 120–145.

[29] Sidky EY Jørgensen JH Pan X . Convex optimization problem prototyping for image reconstruction in computed tomography with the Chambolle–Pock algorithm. Phys Med Biol 2012; 57: 3065–3091. 22538474 10.1088/0031-9155/57/10/3065 PMC3370658

[30] Condat L . A primal-dual splitting method for convex optimization involving lipschitzian, proximable and linear composite terms. J Optim Theory Appl 2013; 158: 460–479.

[31] Vũ BC . A splitting algorithm for dual monotone inclusions involving cocoercive operators. Adv Comput Math 2011; 38: 667–681.

[32] Buades A Coll B Morel JM . A review of image denoising algorithms, with a new one. Mult Model Sim 2005; 4: 490–530.

[33] Wettenhovi VV Hietanen A Niinimäki K , et al. Comparison of three different projectors for cone beam CT. In: *2022 IEEE Nuclear science symposium and medical imaging conference (NSS/MIC)*, 2022. IEEE. DOI: 10.1109/nss/mic44845.2022.10399001.

[34] Galigekere R Wiesent K Holdsworth D . Cone-beam reprojection using projection-matrices. IEEE Trans Med Imag 2003; 22: 1202–1214. 10.1109/TMI.2003.817787 14552575

[35] Jia X Dong B Lou Y , et al. GPU-based iterative cone-beam CT reconstruction using tight frame regularization. Phys Med Biol 2011; 56: 3787–3807. 21628778 10.1088/0031-9155/56/13/004

[36] van Aarle W Palenstijn WJ Beenhouwer JD , et al. The ASTRA toolbox: A platform for advanced algorithm development in electron tomography. Ultramicroscopy 2015; 157: 35–47. 26057688 10.1016/j.ultramic.2015.05.002

[37] Biguri A Dosanjh M Hancock S , et al. TIGRE: a MATLAB-GPU toolbox for CBCT image reconstruction. Biomed Phys Eng Express 2016; 2: 055010.

[38] Zeng G Gullberg G . Unmatched projector/backprojector pairs in an iterative reconstruction algorithm. IEEE Trans Med Imag 2000; 19: 548–555. 10.1109/42.870265 PMC5297459 11021698

[39] Kamphuis C Beekman FJ van Rijk PP , et al. Dual matrix ordered subsets reconstruction for accelerated 3d scatter compensation in single-photon emission tomography. European J Nucl Med Mol Imag 1997; 25: 8–18. 10.1007/s002590050188 9396869

[40] Zeng GL . Counter examples for unmatched projector/backprojector in an iterative algorithm. Chinese J Acad Radiol 2019; 1: 13–24. 10.1007/s42058-019-00006-1 PMC8184118 34104875

[41] Reid EJ Drummy LF Bouman CA , et al. Multi-resolution data fusion for super resolution imaging. IEEE Trans Comput Imag 2022; 8: 81–95.

[42] Lorenz DA Schneppe F . Chambolle-pock’s primal-dual method with mismatched adjoint. *arXiv* 2022; DOI: 10.48550/ARXIV.2201.04928. 2201.04928.

[43] Chouzenoux E Pesquet JC Riddell C , et al. Convergence of proximal gradient algorithm in the presence of adjoint mismatch *. Inverse Prob 2021; 37: 065009.

[44] Chouzenoux E Contreras A Pesquet JC , et al. Convergence results for primal-dual algorithms in the presence of adjoint mismatch. SIAM J Imag Sci 2023; 16: 1–34.

[45] Dong Y Hansen PC Hochstenbach ME , et al. Fixing nonconvergence of algebraic iterative reconstruction with an unmatched backprojector. SIAM J Sci Comput 2019; 41: A1822–A1839.

[46] Elfving T Hansen PC . Unmatched projector/backprojector pairs: Perturbation and convergence analysis. SIAM J Sci Comput 2018; 40: A573–A591.

[47] Hahn K Schöndube H Stierstorfer K , et al. A comparison of linear interpolation models for iterative CT reconstruction. Med Phys 2016; 43: 6455–6473. 27908185 10.1118/1.4966134 PMC5106434

[48] Joseph PM . An improved algorithm for reprojecting rays through pixel images. IEEE Trans Med Imag 1982; 1: 192–196. 10.1109/TMI.1982.4307572 18238275

[49] Siddon RL . Fast calculation of the exact radiological path for a three-dimensional CT array. Med Phys 1985; 12: 252–255. 4000088 10.1118/1.595715

[50] Jacobs F Sundermann E Sutter BD , et al. A fast algorithm to calculate the exact radiological path through a pixel or voxel space. J Comput Inf Technol 1998; 6: 89–94.

[51] Christiaens M De Sutter B De Bosschere K , et al. A fast, cache-aware algorithm for the calculation of radiological paths exploiting subword parallelism. J Syst Archit 1999; 45: 781–790.

[52] Stone JE Gohara D Shi G . OpenCL: A parallel programming standard for heterogeneous computing systems. Comput Sci Eng 2010; 12: 66–73. 21037981 10.1109/MCSE.2010.69 PMC2964860

[53] Alpay A Heuveline V . SYCL beyond OpenCL: The architecture, current state and future direction of hipSYCL. In: *Proceedings of the international workshop on openCL*. IWOCL ’20, ACM. DOI: 10.1145/3388333.3388658.

[54] Lewis J . Fast template matching. Vis Interface 1994; 95: 120–123.

[55] Hensley J Scheuermann T Coombe G , et al. Fast summed-area table generation and its applications. Comput Graph Forum 2005; 24: 547–555.

[56] Nehab D Maximo A Lima RS , et al. GPU-efficient recursive filtering and summed-area tables. ACM Trans Graphics 2011; 30: 1–12.

[57] Yalamanchili P Arshad U Mohammed Z , et al. ArrayFire - A high performance software library for parallel computing with an easy-to-use API, 2015. https://github.com/arrayfire/arrayfire.

[58] Chambolle A Ehrhardt MJ Richtárik P , et al. Stochastic primal-dual hybrid gradient algorithm with arbitrary sampling and imaging applications. SIAM J Optim 2018; 28: 2783–2808.

[59] Tang J Ehrhardt M Schönlieb CB . Stochastic primal-dual three operator splitting with arbitrary sampling and preconditioning. *arXiv* 2022. DOI: 10.48550/ARXIV.2208.01631. 2208.01631.

[60] Beck A Teboulle M . A fast iterative shrinkage-thresholding algorithm for linear inverse problems. SIAM J Imag Sci 2009; 2: 183–202.

[61] Zhang H Zeng D Zhang H , et al. Applications of nonlocal means algorithm in low-dose X-ray CT image processing and reconstruction: A review. Med Phys 2017; 44: 1168–1185. 28303644 10.1002/mp.12097 PMC5381744

[62] Chen Y Gao D Nie C , et al. Bayesian statistical reconstruction for low-dose x-ray computed tomography using an adaptive-weighting nonlocal prior. Comput Med Imag Graphics 2009; 33: 495–500. 10.1016/j.compmedimag.2008.12.007 19515533

[63] Zhang H Ma J Wang J , et al. Statistical image reconstruction for low-dose CT using nonlocal means-based regularization. Comput Med Imag Graphics 2014; 38: 423–435. 10.1016/j.compmedimag.2014.05.002 PMC4152958 24881498

[64] Kelm ZS Blezek D Bartholmai B , et al. Optimizing non-local means for denoising low dose CT. In: *2009 IEEE international symposium on biomedical imaging: from nano to macro*, 2009. IEEE. DOI: 10.1109/isbi.2009.5193134.

[65] Giraldo JCR Kelm ZS Guimaraes LS , et al. Comparative study of two image space noise reduction methods for computed tomography: Bilateral filter and nonlocal means. In: *2009 Annual international conference of the ieee engineering in medicine and biology society*, 2009. pp.3529–3532. DOI: 10.1109/IEMBS.2009.5334714. 19964998

[66] Li Z Yu L Trzasko JD , et al. Adaptive nonlocal means filtering based on local noise level for CT denoising. Med Phys 2014; 41: 011908. 24387516 10.1118/1.4851635

[67] Chen Y Yang Z Hu Y , et al. Thoracic low-dose CT image processing using an artifact suppressed large-scale nonlocal means. Phys Med Biol 2012; 57: 2667. 22504130 10.1088/0031-9155/57/9/2667

[68] Segars WP Sturgeon G Mendonca S , et al. 4D XCAT phantom for multimodality imaging research. Med Phys 2010; 37: 4902–4915. 20964209 10.1118/1.3480985 PMC2941518

[69] Badal A Badano A . Accelerating monte carlo simulations of photon transport in a voxelized geometry using a massively parallel graphics processing unit. Med Phys 2009; 36: 4878–4880. 19994495 10.1118/1.3231824

[70] Li T Feng H Xu Z , et al. Comparison of different analytical edge spread function models for MTF calculation using curve-fitting. In: Maître H, Sun H, Lei B et al. (eds) *MIPPR 2009: Remote Sensing and GIS data processing and other applications*, 2009. SPIE. DOI: 10.1117/12.832793.

[71] Wang Z Bovik AC Sheikh HR , et al. Image quality assessment: From error measurement to structural similarity. IEEE Trans Image Process 2004; 13: 600–612. 15376593 10.1109/tip.2003.819861

[72] Wang Z Bovik AC . Mean squared error: Love it or leave it? IEEE Signal Process Mag 2009; 26: 98–117.

